# Relationship Between Perceived Stress and Anxiety in High School Senior Students: The Mediating Role of Social Support and the Moderating Influence of Lifestyle

**DOI:** 10.3390/healthcare14020263

**Published:** 2026-01-21

**Authors:** Vilija Malinauskiene, Romualdas Malinauskas

**Affiliations:** Department of Physical and Social Education, Lithuanian Sports University, Sporto 6, LT-44221 Kaunas, Lithuania

**Keywords:** anxiety, protective and risk factors, social support, lifestyle, high school senior students

## Abstract

(1) Background: The purpose of this study was to investigate the associations between perceived stress and anxiety in high school senior students, taking into account the possible influence from lifestyle (physical activity, nicotine dependence, and alcohol use) and social support. (2) Methods: A representative sample of high school senior students (N = 405; mean age: 18.2 ± 0.4), reflecting the overall geographic distribution of Lithuania’s student population, was investigated using anonymous questionnaires on perceived stress (Perceived Stress Scale, PSS-10), anxiety (Generalized Anxiety Disorder Scale, GAD-7), perceived social support (Multidimensional Scale of Perceived Social Support, MSPSS), lifestyle (Fagerström Test for Nicotine Dependence), alcohol use (Alcohol Use Disorders Identification Test, AUDIT), and physical activity (International Physical Activity Questionnaire, IPAQ). Hierarchical multiple regression analysis was employed, investigating mediating and moderating effects. (3) Results: The direct effect of perceived stress on anxiety was significant (*B* = 0.364; SE = 0.0486; 95% CI [0.268, 0.459]; *p* < 0.001). Furthermore, the analysis revealed a significant indirect effect via social support (*B* = 0.387; SE = 0.0525; 95% CI [0.284, 0.490]), indicating that a portion of the relationship between perceived stress and anxiety was mediated by social support. Physical inactivity, alcohol use, and nicotine dependence served as moderators. Our findings confirmed that all three moderators showed significant interaction effects, with standardized β = −0.124, *p* = 0.002, for physical inactivity, β = 0.073, *p* = 0.016, for alcohol dependence, and β = 0.119, *p* = 0.001, for nicotine dependence, in the relationship between perceived stress and anxiety among high school senior students. (4) Conclusions: These findings have practical insights for educators who implement physical activity and alcohol and nicotine usage programs for high school senior students to help reduce their stress and anxiety.

## 1. Introduction

Stress and anxiety among high school students is a growing concern worldwide. These emotional and psychological responses (academic pressure, future uncertainty, peer pressure and social issues, parental expectations, etc.) can significantly impact students’ academic performance, social relationships, and overall well-being [1]. Heightened academic stress in the final years of schooling is a common concern. Importantly, the present study focuses on perceived stress, which is conceptualized as the degree to which individuals appraise situations in their lives as unpredictable, uncontrollable, or overwhelming [1]. Thus, perceived stress reflects a cognitive appraisal process, rather than an emotional response per se. Perceived stress is conceptualized as a prior cognitive process that increases the likelihood of anxiety symptoms.

Adolescence is a period of extreme change. A growing body of research has established a clear association between stress and anxiety during this life stage, whereby adolescent stress functions as a predictor of adverse mental health outcomes, including anxiety [2]. In this context, anxiety is defined as a pervasive emotional state marked by excessive worry, apprehension, and physiological tension, often triggered by perceived threats and capable of significantly impairing daily functioning [2]. Anxiety among high school students can affect school performance, relationships, and family life. A systematic review of 61 articles found significant increases in the rates of anxiety among children and adolescents [3]. Furthermore, recent reviews highlight the multifaceted nature of adolescent anxiety, identifying psychosocial stress and insufficient social support as key contributing factors [4]. Anxiety remains one of the most prevalent psychiatric conditions among adolescents and young people [5], with symptoms including sleep disturbances, somatic complaints, irritability, concentration difficulties, fatigue, social withdrawal, and poor academic performance [5]. Although anxiety among high school seniors is a significant and impactful concern, appropriate support systems and effective coping mechanisms can help students manage these challenges more successfully [6]. Thus, in this study, perceived stress is treated not as a synonym for anxiety but as a separate cognitive construct reflecting a person’s assessment that the demands of the environment exceed their coping resources and acting as a predictor of anxiety symptoms.

Studies often highlight the protective role of social support for anxiety [7,8]. The present study focuses on social support, a multidimensional concept that could be characterized as the perception and intentionality of being cared for and supported by others (family, friends, and other significant people) [8]. Investigations on stress, anxiety, and social support show various directions: (a) studies in which social support predicts anxiety, (b) studies in which social support functions as a mediator, (c) studies in which social support acts as a moderator, and (d) studies examining stress → anxiety pathways.

Studies have consistently demonstrated that social support plays a significant role in predicting anxiety [6,7], and higher levels of social support are correlated with reduced anxiety levels. Social support significantly reduces anxiety by acting as a buffer against negative impacts, promoting positive lifestyles, and enhancing resilience. It provides emotional comfort, offers practical help, and fosters a sense of belonging, reducing feelings of isolation and improving overall mental well-being. As identified in a recent study [7], social support is an important protective factor for lower anxiety levels: students who report a higher sense of peer and family support report less anxiety. Connecting with friends, family, or professionals creates a safe space in which to share feelings and gain perspective, making it a crucial strategy for managing life’s challenges.

Several empirical studies have specifically identified social support as a mediator between stress and anxiety [8,9]. Social support allows stressful situations and coping strategies to be redefined, protecting individuals from the consequent negative effects (anxiety) [8]. Recent research examined social support mediating academic stress and anxiety in university students [8], and it was established that social support significantly and positively mediates the relationship between academic stress and anxiety in men. Another study aimed at exploring whether perceived stress mediates the relationship between social support and anxiety [9].

Other researchers have examined social support as a moderator of the stress–anxiety relationship. For instance, studies investigating early adolescents tested social support as both a mediator and a moderator between perceived stress and anxiety symptom patterns [10]. Using multiple regression analysis procedures specified for the testing of moderation and mediation, results indicated that social support did not play a moderating role in the relationship between perceived stress and symptom patterns of anxiety, but social support did play a mediating role in this relationship [10].

It is important to highlight an additional research area that focuses on the pathways between stress and anxiety [7,9,11]. For example, one prior study utilized Lazarus and Folkman’s stress and coping theory [9], while another employed the stress-buffering hypothesis to investigate the mediating effect of social support on the connection among perceived stress, various aspects of social support, and anxiety [11]. The stress-buffering hypothesis indicates that social support positively impacts individuals’ well-being by increasing positive emotions and enhancing perceived self-esteem (main effect) [11]. Nonetheless, social support can also enhance individuals’ well-being indirectly by mitigating stressful circumstances or by diminishing the effects of stressful experiences (buffering effect) [11].

**Figure 1:**
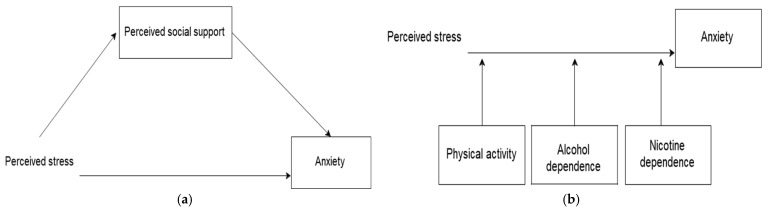
The theoretical framework models: (**a**) mediation model; (**b**) moderation model.

In the present study, we draw on Lazarus and Folkman’s stress and coping theory, highlighting the importance of social support in shaping individuals’ perceptions of stress [12]. An event is deemed stressful when its demands surpass available resources, increasing stress levels. Conversely, if individuals view an event as manageable and believe that they possess the necessary resources, their stress levels are likely to diminish [12]. According to stress and coping theory, social support can facilitate the use of adaptive coping strategies (like seeking emotional support) and can lessen anxiety in stressful situations [12]. Thus, in the present study, perceived stress is treated as a conceptually distinct antecedent of anxiety, while social support is expected to influence how perceived stress is transformed into anxiety (Figure 1a).

Considering Lazarus and Folkman’s theory of stress and coping, many adolescents can resort to various coping strategies to manage stress [12]. While some use adaptive strategies (like problem-solving or seeking support), others turn to maladaptive lifestyle habits such as physical inactivity, alcohol consumption and/or smoking (Figure 1b) [13]. We defined lifestyle as a set of behavioral patterns (habits) that impact health outcomes (in our study, anxiety) [13].

For instance, alcohol is often used to alleviate anxiety, but it can paradoxically worsen psychological distress over time [13]. As students’ anxiety may arise from or alongside stress, it contributes to emotional dysregulation, often leading to unhealthy lifestyle habits like alcohol use and smoking. Social anxiety is recognized as a risk factor for alcohol use disorders among college students [14]. Research indicates that adolescents may turn to alcohol as a coping mechanism when faced with stress. Other researchers found that individuals with high levels of perceived stress were more likely to use alcohol to manage their feelings, which can create a false sense of relief [15]. Conversely, regular alcohol use can impair the body’s stress response system, leading to increased baseline anxiety levels over time. After the effects of alcohol wear off, individuals might experience heightened anxiety, intensifying the cycle. Behavioral research demonstrates that drinking to cope with negative affect is a potent marker for current and future problems with alcohol [15]. Yet another study has shown that chronic alcohol use leads to adaptive changes in these systems, potentially resulting in increased anxiety levels when not drinking [16]. Research also indicates that alcohol dependance is strongly linked to poor academic performance, risky behaviors, and anxiety [17]. A longitudinal study tracked participants over several years and found that those who used alcohol as a stress response were more likely to develop anxiety disorders [18]. The study suggested that this relationship may be bidirectional; while stress leads to increased alcohol use, higher anxiety levels can also drive individuals to seek alcohol as a way to cope [18]. Although previous studies have established that stress predicts anxiety and that alcohol use is associated with both, the moderating role of alcohol use in the stress–anxiety link has not been widely examined among adolescents. Exploring this moderation can help determine whether alcohol use strengthens the association between stress and anxiety—that is, whether adolescents who drink experience more anxiety when stressed than those who do not drink.

Even though social anxiety appears to be a risk factor for smoking and nicotine dependence, little work has identified factors that may play a role in these relationships [19]. A systematic review of anxiety across smoking stages in adolescents and young adults reinforced anxiety as a significant risk factor for smoking in one’s lifetime [20]. Smoking during adolescence is positively associated with increased anxiety symptoms, controlling for socioeconomic status and other factors, acting as an exogenous risk factor in anxiety development [21]. On the other hand, stress levels are higher in adolescent smokers compared to non-smokers, and smoking often serves as a maladaptive coping mechanism to deal with stress and anxiety symptoms [22].

In addition to ongoing research on alcohol and smoking associations with adolescents’ mental health, investigations in recent years addressed physical activity among adolescents and its associations with mental health [23]. These studies, presented in a systematic review and meta-analysis, confirm that physical activity/sport as a lifestyle factor during adolescence could be an adaptive coping strategy in the stress–anxiety relationship [23]. Recent studies among adolescents found that physical activity can buffer the effects of stress and that physical activity suppresses the negative impact of stress on the mental health of adolescents [24,25]. Moreover, physical activity serves as a buffer that can moderate the link between stress and anxiety in adolescents, meaning that it can reduce the impact of stress on anxiety levels [26,27]. The effect of physical activity on mental health is multifactorial: regular exercise enhances emotion regulation [28], boosts self-efficacy [29], and stimulates endorphin release [30], all of which contribute to lower anxiety and better coping with stress. Therefore, physical activity can protect adolescents’ mental health by mitigating the negative effects of stressors. A study examining the interplay between physical activity, problematic internet use, and the negative emotional states of depression, anxiety, and stress highlighted the role of physical activity moderation in the association between problematic internet use and negative emotional state outcomes [30].

It is important to note that no explicit typology (e.g., clusters) fully categorizes 17–19-year-old students in Lithuania by combined physically active lifestyle, alcohol, and nicotine use, but inverse patterns emerge: physically active students engage less in smoking and drinking than sedentary peers [31]. Recent HBSC trends highlight declining substance use alongside stable low physical activity, suggesting potential “healthy” (active; low substance use) and “risky” (inactive; higher use of electronic cigarettes) subgroups, with gender (boys being more active/smokers) and age gradients [31].

### Aims and Hypotheses

This study aimed at investigating the predictors of anxiety in a sample of adolescents. The first objective was to show the effect sizes of perceived stress, social support, and lifestyle (alcohol and nicotine dependence and physical inactivity) as independent variables on the dependent variable—anxiety—and to establish whether these independent variables allow us to predict the associations between perceived stress and anxiety. The second objective was to investigate whether social support acted as a mediator in the associations between perceived stress and anxiety among adolescents. The third objective was to assess whether lifestyle factors (alcohol and nicotine dependence; physical inactivity) acted as moderators between perceived stress and anxiety.

We decided to test two different models—one for mediation and one for moderation—because this approach is theoretically sound and methodologically advantageous for several reasons. Firstly, combining complex mediation and moderation effects into a single, intricate model can lead to difficulties in interpretation. Each analysis, when conducted separately, provides a clearer, more focused understanding of its respective mechanism [32]. This approach prevents conflating the “explanation” of an effect (mediation) with the “contingency” of an effect (moderation) [32]. Secondly, including numerous interaction terms (for moderation) within a complex mediation model can substantially increase model complexity, demand larger sample sizes, and potentially reduce statistical power for detecting specific effects, especially if some interactions are not theoretically well-grounded. Separating them allows for more parsimonious models tailored to testing specific hypotheses, improving the robustness of each set of findings [33]. Thirdly, a sequential approach to theoretical advancement is often employed. Researchers typically first establish a foundational relationship (e.g., stress–anxiety), then explore the mechanisms (mediation) that explain it, and subsequently investigate the conditions (moderation) under which that relationship or its mechanisms operate more or less strongly [34]. This sequential approach reflects a logical progression of inquiry.

Based on Lazarus and Folkman’s theory, and past empirical research findings [6,7,8,9,10,11,12], in this study, we hypothesized (H1) that perceived stress, social support, and lifestyle factors are predictors of anxiety. The second hypothesis of our study (H2) was that social support serves as a mediator between stress experience and anxiety among high school senior students (Figure 1a). Mediation implies that social support helps explain the relationship between perceived stress and anxiety but does not indicate that social support influences the strength or direction of the relationship between perceived stress and anxiety. According to stress and coping theory, individuals utilize social support as a coping mechanism [12]. When individuals perceive stress, the availability of social support can influence their coping strategies. For example, having someone to talk to or seek advice from can help individuals reframe their stressful situations, reducing anxiety (i.e., social support mediates the impact of perceived stress on anxiety, as it shapes the coping process).

Coping theory, developed by Lazarus and Folkman, posits that individuals evaluate and respond to stressors through cognitive appraisal and the selection of coping strategies [12]. Considering Lazarus and Folkman’s theory of stress and coping and the previously mentioned assumptions, we included lifestyle (alcohol and nicotine dependence, physical inactivity) into our study and our third hypothesis (H3) was that lifestyle factors (alcohol and nicotine dependence; physical inactivity) acted as moderators in the associations between stress and anxiety among adolescents (Figure 1b). We tested lifestyle factors including alcohol and nicotine dependence, and participation in physical exercise, which are integral to the improvement of individual’s health [35]. According to Lazarus and Folkman’s theory, our hypothesis was that lifestyle factors (alcohol and nicotine dependence; physical inactivity) used as maladaptive coping strategies reinforce the positive relationship between stress and anxiety. In the present study, we are trying to show that physical inactivity and alcohol and nicotine dependence are independent moderators between stress and anxiety on the basis of Lazarus and Folkman’s theory of stress and coping [12], which recognizes that associations between stress and anxiety are influenced by lifestyle moderators (alcohol and nicotine dependence; physical inactivity) that play a pivotal role in enhancing an individual’s health [35].

Moderation was tested only in the direct relationship between stress and anxiety, because theoretically, a moderator is considered to be a factor that changes an individual’s response to stress, rather than the occurrence of stress or other psychological processes. Therefore, it was expected that moderators would determine the strength of the effect of stress on anxiety, but not other relationships in the model. According to Lazarus and Folkman’s theory of stress and coping [12], anxiety can be interpreted as a direct emotional response to subjectively assessed stress, while individual behavioral and lifestyle factors act as resources or vulnerabilities that determine the intensity of this response. Alcohol and nicotine dependence, and physical inactivity are theoretically considered factors that weaken adaptive stress coping and increase emotional reactivity, so they were modeled as moderators in the direct relationship between stress and anxiety.

## 2. Materials and Methods

### 2.1. Study Design

A quantitative, cross-sectional design was employed. This design is suitable for assessing relationships among variables at a single point in time and testing mediation and moderation hypotheses.

### 2.2. Study Participants and Procedure

Participants were selected using a stratified random sampling method, ensuring balanced representation by region and school type (gymnasiums and basic schools). Regional representation included Vilnius County (20%), Kaunas County (17%), Klaipėda County (11%), and other counties such as Šiauliai, Panevėžys, Alytus, and Utena comprising the remaining 52%, reflecting the general geographic distribution of Lithuania’s student population. The study was conducted in a representative sample of 470 last-grade high school students from various regions of Lithuania. Sample size was estimated to be approximately 385 participants (based on the G*Power version 3.1 program for medium effect size; α = 0.05; power = 0.80). In total, 405 students filled in the questionnaires, giving a response rate of 86.17%. Of the participants, 63.2% were female (n = 256) and 36.8% were male (n = 149). The mean age of the students was 18.2 years (SD = 0.4), with an age range from 17 to 19 years. In terms of school type, 67% attended gymnasiums, while 33% were enrolled in basic schools. Students outside this age range or those not currently attending high school were excluded, as were individuals whose data was incomplete or who declined to participate.

Approval from the Ethics Committee of the university (Protocol No. SMTEK-17) was obtained, as were consent forms from schools, parents, and participants. The online questionnaires were administered during school hours. The anonymity and confidentiality of the responses were ensured because no personal information was collected.

### 2.3. Perceived Stress (PSS-10)

PSS-10 (Perceived Stress Scale) was applied to measure the stress levels of participants. Developed by Sheldon Cohen in 1983, it is a widely recognized tool for measuring the perception of stress in individuals [36]. This scale asks respondents about their feelings and thoughts over the past month, utilizing a 5-point Likert scale (0 = Never; 1 = Almost Never; 2 = Sometimes; 3 = Fairly Often; 4 = Very Often) to gauge the frequency of stress-related experiences. The PSS-10 score is then obtained by summing across all items. Higher scores indicate higher levels of perceived stress. A Lithuanian adaptation of the questionnaire has been performed [37]. The adaptation process involved translation, back-translation, and cultural adaptation to ensure that the meaning of the original statements was maintained [37].

### 2.4. Generalized Anxiety Disorder Scale (GAD-7)

Anxiety symptoms were assessed using the Generalized Anxiety Disorder Scale (GAD-7) [38]. This 7-item scale measures anxiety severity on a scale from 0 (not at all) to 4 (nearly every day). Total scores categorize anxiety levels as 0–4 (none), 5–9 (mild), 10–14 (moderate), and 15–21 (severe). A Lithuanian adaptation of the questionnaire has been performed [39].

### 2.5. Multidimensional Scale of Perceived Social Support (MPSSS)

The Multidimensional Scale of Perceived Social Support (MPSSS), developed by Zimet, comprises 12 items measuring subjective perceptions of support from family, friends, and others [40,41]. Responses range from 1 (very strongly disagree) to 7 (very strongly agree) using Likert scaling. An example item includes: “I can rely on my friends when things go wrong.” Higher scores indicate greater perceived social support. The questionnaire has been adapted for use in Lithuania [42].

### 2.6. International Physical Activity Questionnaire (IPAQ Short)

The IPAQ short form asks about three specific types of activity undertaken, namely, walking, moderate-intensity activities, and vigorous-intensity activities. Participants report the number of days per week and the number of minutes per day that they spent on each activity. The score for each type of physical activity in metabolic equivalent intensity in minutes per week (MET-min/week) is calculated by multiplying the number of minutes per week devoted to each category of activity by the MET value of this activity (walking = 3.3 × minutes of walking × days of walking; moderate activity = 4.0 × minutes of moderate activity × days of moderate activity; vigorous activity = 8.0 × minutes of vigorous activity × days of vigorous activity). A combined total physical activity MET-min/week was calculated. Based on the scoring protocol provided by the IPAQ [43], levels of physical activity can be divided into three categories: insufficiently active (0–599 MET-min/week), moderately active (600–2999 MET-min/week), and health-enhancing physically active (3000 or more MET-min/week). Logarithmic transformation was applied to the scores. The questionnaire has been translated and culturally adapted for use in Lithuania [44].

### 2.7. Alcohol Use Disorders Identification Test (AUDIT)

The Alcohol Use Disorders Identification Test (AUDIT) (self-report version) is a comprehensive 10-question alcohol harm screening tool [45]. It was developed by the World Health Organization (WHO) and has been used in a variety of health and social care settings; it has also been adapted for use in Lithuania [46]. The 10-question self-report tool assesses alcohol dependence. Responses to the 10 questions are scored on a 0–4 scale, giving a total possible score of 40. Scores are categorized to indicate risk levels, with higher scores suggesting greater risk of problematic alcohol use (0–7: low risk or abstinence; 8–15: hazardous or harmful alcohol use; 16 or more: alcohol dependence).

### 2.8. Fagerström Test for Nicotine Dependence (FTND)

The Fagerström Test for Nicotine Dependence (FTND) was used to assess the level of smokers’ nicotine dependence [47]. The FTND questionnaire consists of 6 questions, each of which is designed to assess various aspects of nicotine dependence. After data collection, responses were analyzed to calculate the FTND scores for each participant. The level of nicotine dependence was categorized into low, moderate, and high. A Lithuanian adaptation of the questionnaire has been performed [48].

### 2.9. Statistical Analysis

All statistical analyses were conducted using SPSS (Version 29.0) including R software—Version 4.2.0) and the mediation package for causal mediation analysis [49,50]. Descriptive statistics of data are presented. Skewness and kurtosis coefficients were computed for univariate normality analyses purposes, and all values were within ±2 (Table 1). To test gender differences, Student’s *t*-test was performed; because the data were normally distributed, Cohen’s d was evaluated. Cohen’s d is a standardized measure of the effect size that quantifies the difference between two population means, expressed in terms of the pooled standard deviation. It helps determine the magnitude of the difference between groups, with larger values indicating a greater difference [51]. Correlations were presented as Pearson product moment correlations (two-tailed) for all continuous variables. The requirement for the use of the linear regression of the multicollinearity of the predictors was verified because correlation values between independent variables above 0.8 might suggest a multicollinearity problem [52]. To examine the predictive value of perceived stress and social support as the independent variables of anxiety, hierarchical multiple regression analyses were executed with the components of lifestyle (physical inactivity, alcohol dependence, and nicotine dependence). We tested the moderation effects of lifestyle factors (physical inactivity, alcohol dependence, and nicotine dependence) as well as perceived social support in the associations between perceived stress and anxiety in the investigated sample using hierarchical moderated regression analyses. The predictor variables (main effects) were entered into the regression equation in Block 1, followed by the 2-way interactions in Block 2. The independent variables were centered by standardizing them before the product term was created [53]. The standardized solution was then examined. We analyzed the strength of the moderating effect of lifestyle factors, calculated the simple slopes, and plotted a moderation effect diagrams. The effect sizes were calculated as r. We tested the mediation model that social support serves as a mediator between stress experience and anxiety among high school senior students using the mediation package for causal mediation analysis [50]. We reported the 95% confidence intervals based on 1000 bootstrap samples.

## 3. Results

### 3.1. Descriptive and Correlational Analysis

Table 1 presents the scales’ means, the standard deviations for the total sample, skewness, kurtosis, and Cronbach α.

Table 2 presents gender differences, with girls prevailing in perceived social support and boys in anxiety, nicotine and alcohol dependence, and physical activity, while differences in perceived stress were insignificant. Table 3 presents Pearson correlations between study variables; the majority of them were significantly correlated, except perceived stress and physical activity. Some correlations were definitely high. It is essential that the independent variables are not too highly correlated with each other, a condition known as multicollinearity. In this sample, correlation coefficients are below 0.80, which indicates the lack of multicollinearity [52].

### 3.2. Hierarchical Multiple Regression Analysis

We performed hierarchical multiple regression analysis with anxiety as the dependent variable and gender and perceived stress as the dependent variables in Block 1, with a definitely high R^2^ 0.288 of the model (Table 4). The standardized β for perceived stress was 0.466. In Block 2, we included social support, and standardized β for perceived stress diminished to 0.236, indicating the mediating effect of social support. The standardized β for social support in the model was −0.673 and statistically significant. In Block 3, we included alcohol dependency, and the ΔR^2^ of the model was 0.47 and significant, while the standardized β for social support diminished to −0.394 and remained significant. The standardized β for perceived stress remained stable and significant, while gender did not show significant effects, though in the Pearson‘s correlation matrix, it correlated significantly with almost all variables. In Block 4 of the hierarchical multiple linear regression, we added nicotine dependence and physical activity as independent variables; the ΔR^2^ of the model increased slightly but remained significant. This model revealed perceived stress, social support, alcohol dependence, nicotine dependence, and physical activity as predictors of anxiety.

### 3.3. Mediation Analysis

A mediation analysis was conducted to assess the relationship between perceived stress, as measured by the PSS-10, and anxiety, as measured by the GAD-7, with social support from the MSPSS as the mediator (Table 5). The total effect of perceived stress on anxiety was significant (*B* = 0.751; SE = 0.0671; *p* < 0.001). The direct effect of perceived stress on anxiety was also significant (*B* = 0.364; SE = 0.0486; 95% CI [0.268, 0.459]; *p* < 0.001). Furthermore, the analysis revealed a significant indirect effect via MSPSS (*B* = 0.387; SE = 0.0525; 95% CI [0.284, 0.490]), indicating that a portion of the relationship between perceived stress and anxiety was mediated by social support. The components of the mediation analysis showed that the path from perceived stress to MSPSS was significant (*B* = −1.609; SE = 0.2058; *p* < 0.001), as was the path from MSPSS to anxiety (*B* = −0.240; SE = 0.0109; *p* < 0.001).

### 3.4. Moderation Analysis

Our results indicated that all three moderators showed significant interaction effects, with standardized β = −0.124, *p* = 0.002, for physical activity, β = 0.073, *p* = 0.016, for alcohol dependence, and β = 0.119, *p* = 0.001, for nicotine dependence (Table 6). All three interaction models were tested for statistical significance and showed significant ΔR^2^ (ΔR^2^ = 0.015, *p* < 0.01 for Interaction Z_physical activity × Z_perceived stress; ΔR^2^ = 0.005, *p* < 0.05 for Interaction Z_alcohol dependence × Z_perceived stress; ΔR^2^ = 0.013, *p* < 0.001 for Interaction Z_nicotine dependence × Z_perceived stress; “Z” indicates that the variable has been standardized).

**Figure 2:**
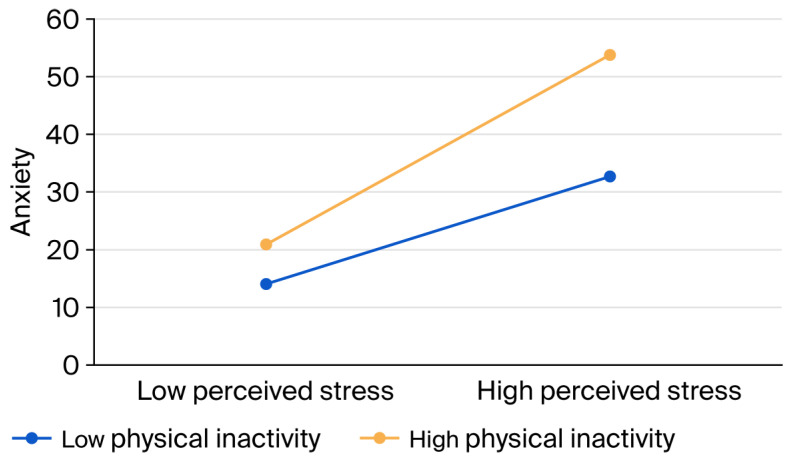
The moderation effect diagram illustrating the relationship between physical inactivity and the dependent variable (anxiety) across low and high levels of perceived stress.

Further analysis of the moderating effect of physical activity intensity showed (Figure 2) that for individuals with high physical inactivity, the slope was statistically significant: *B_simple_* = 0.72; *t*(401) = 2.93; *p* = 0.004; r = 0.15. According to Cohen’s guidelines, r = 0.15 is considered a small effect size [51]. For individuals with low physical inactivity, the slope was also statistically significant (*B_simple_* = 1.27; *t*(401) = 2.62; *p* = 0.009; r = 0.13), indicating a small effect size according to Cohen’s criteria. Overall, the results indicate that perceived stress is a statistically significant predictor of anxiety in both low- and high-physical-activity groups. However, the strength of the relationship between perceived stress and anxiety was modest in both cases, with slightly stronger effects observed among individuals with high physical inactivity compared to those with low physical inactivity.

**Figure 3:**
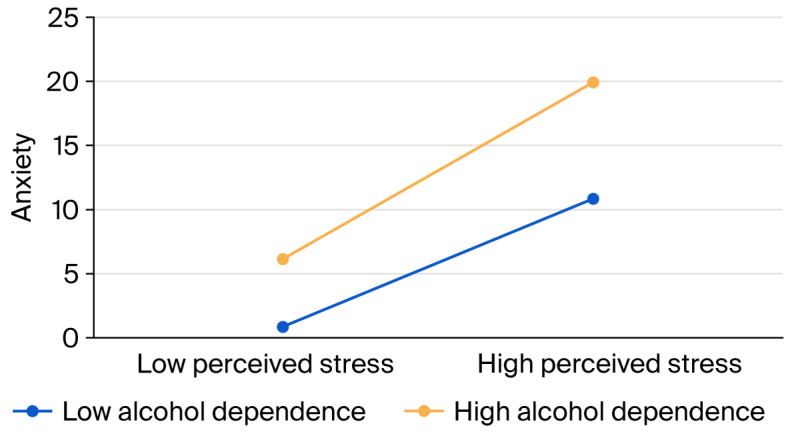
The moderation effect diagram illustrating the relationship between alcohol dependence and the dependent variable (anxiety) across low and high levels of perceived stress.

Further analysis of the moderating effect of alcohol dependence showed (Figure 3) that for individuals with low alcohol dependence, perceived stress was a significant positive predictor of anxiety, such that higher levels of perceived stress were associated with greater anxiety: *B_simple_* = 0.37; *t*(401) = 2.10; *p* = 0.036. The magnitude of this association, represented by the correlation coefficient, was r = 0.10, indicating a small effect size according to Cohen’s guidelines [51]. For individuals with high alcohol dependence, perceived stress also significantly predicted anxiety: *B_simple_* = 0.51; *t*(401) = 2.20; *p* = 0.028. The effect size was calculated as r = 0.11, likewise reflecting a small effect.

The data indicate that while perceived stress is a significant predictor of anxiety in both low- and high-alcohol-dependence groups, the strength of the relationship (as indicated by small effect sizes) is greater for students with high alcohol dependence compared to those with low alcohol dependence.

**Figure 4:**
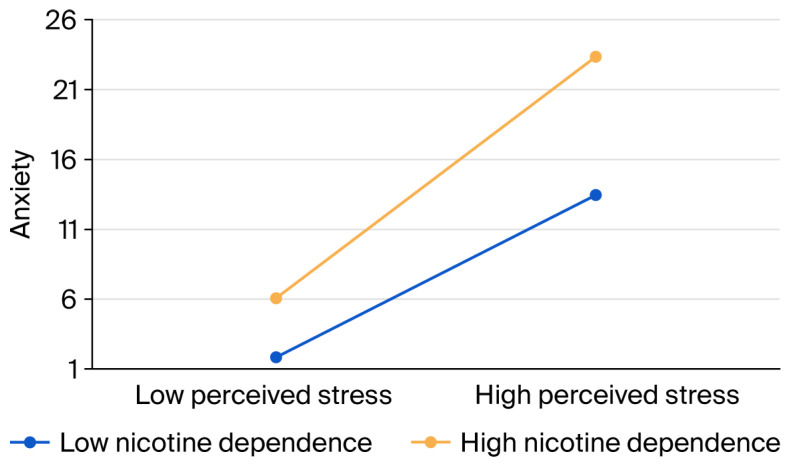
The moderation effect diagram illustrating the relationship between nicotine dependence and the dependent variable (anxiety) across low and high levels of perceived stress.

As the results in Table 6 show a highly significant interaction term “Z_Nicotine Dependence (FTND) × Z_Perceived Stress”, suggesting a moderation effect, we further analyzed the strength of the moderating effect (Figure 4). For individuals with low nicotine dependence, perceived stress was a significant positive predictor of anxiety, such that higher levels of perceived stress were associated with greater anxiety: *B_simple_* = 0.43; *t*(401) = 6.89; *p* < 0.001. The magnitude of this correlation effect, represented by the correlation coefficient, is r = 0.33. According to Cohen’s guidelines, r = 0.33 is considered a medium–strong effect [51]. For individuals with high nicotine dependence, perceived stress also significantly predicted anxiety: *B_simple_* = 0.64; *t*(401) = 7.16; *p* < 0.001. The effect size was calculated as r = 0.34, and also reflects a medium–strength effect. The data indicate that, while perceived stress is a significant predictor of anxiety in both low- and high-nicotine-dependence groups, the relationship (as indicated by medium effect sizes) is stronger for students with high nicotine dependence compared to those with low nicotine dependence.

The moderation analyses revealed that the positive relationship between perceived stress and anxiety is stronger for students with lower physical activity, with more alcohol dependence, and with more nicotine dependence. Therefore, we can conclude that physical activity is a protective factor in the associations between perceived stress and anxiety in adolescents; that is, physical activity weakens the stress–anxiety relationship. Our results confirm that alcohol and nicotine dependence and physical inactivity act as moderators with significant effects.

## 4. Discussion

This study contributes to the growing body of literature on student mental health by examining the interrelationships among stress, anxiety, social support, alcohol and smoking dependence, and physical activity. We investigated the associations between perceived stress and anxiety among senior adolescents in a representative sample of Lithuanian high schools. Our first hypothesis (H1) was confirmed because we found that perceived stress, social support, and lifestyle are predictors of anxiety, and our findings are consistent with Lazarus and Folkman’s theory and other investigations among adolescents [11,54,55].

We also tested whether social support acts as a mediator (H2) between perceived stress and anxiety among adolescents, and found that social support is a mediator but not a moderator, highlighting the importance of this particular variable. In the context of Lazarus and Folkman’s theory of stress and coping, adolescents leverage social support as a coping strategy [12]. When faced with stress, the presence of social support can affect how they approach coping. For instance, having someone to confide in or consult for advice can assist individuals in reinterpreting their stressful circumstances, thereby alleviating anxiety. This means that social support mediates the impact of perceived stress on anxiety, as it shapes the coping process. Though scientific evidence is inconsistent, with investigations both denying and confirming the role of social support as a moderator in the associations between perceived stress and anxiety [7,12,56], our findings support the mediation hypothesis and the statement that adolescents should increase their social ties and gain support in everyday life from peers, family, and school.

We tested our third hypothesis (H3) that lifestyle factors (alcohol and nicotine dependence; physical inactivity) acted as moderators in the associations between stress and anxiety among adolescents in the investigated sample, and found a significant effect for all three lifestyle factors. Our study confirmed that adolescents choose alcohol and smoking when exposed to stress and anxiety in their everyday life. In adolescents, two main categories dominate drinking motives, namely, the belief that alcohol will enhance positive affect (enhancement) and the belief that alcohol will help cope with negative affect (coping) [57]. Research indicates that adolescents may turn to alcohol as a coping mechanism when faced with stress. Other studies confirm that individuals with high levels of perceived stress are more likely to use alcohol to manage their feelings, which can create a false sense of relief, and that moderate alcohol use might temporarily reduce feelings of stress and anxiety, acting as a short-term coping mechanism [15]. This can create a perception that alcohol alleviates stress-related anxiety [15,58]. Conversely, regular alcohol use can impair the body’s stress response system, leading to increased baseline anxiety levels over time [59]. After the effects of alcohol wear off, individuals might experience heightened stress and anxiety, intensifying the cycle; heavy drinking could strengthen the link between stress and anxiety, making individuals more vulnerable to anxiety disorders [60]. Our study confirmed that alcohol dependence is a moderator between perceived stress and anxiety among adolescents in the direction that those with alcohol dependence experience more anxiety when exposed to stress. Other studies [59,61] confirm that alcohol’s role as a moderator is complex. It may temporarily dampen the stress–anxiety link in some individuals but can also reinforce or worsen anxiety over time, especially with heavy or chronic use [59,61].

Smoking among adolescents is associated with worse mental health outcomes, including higher anxiety symptoms, depressive symptoms, suicidal ideation, and poorer school performance. Cigarette smoking is often used as a maladaptive coping strategy to relieve tension and anxiety, despite its detrimental physical and psychological effects [62]. Our study confirmed that adolescents with more nicotine dependence experience more anxiety when exposed to stress. There is some evidence that adolescents with anxiety sensitivity (heightened sensitivity to anxious arousal) who smoke report the highest levels of anxiety [63], suggesting that smoking amplifies anxiety vulnerability. Smoking is linked to increased anxiety symptoms characterized by physiological arousal, indicating a bidirectional relationship where anxiety symptoms and smoking reinforce each other [64]. Smoking acts as a moderator in the relationship between stress and anxiety in adolescents by intensifying anxiety symptoms and emotional vulnerabilities. It appears that smoking not only responds to stress and anxiety but also exacerbates these mental health challenges, especially for vulnerable adolescent populations [65]. This understanding highlights the importance of targeting smoking prevention and cessation in adolescents as part of mental health interventions addressing stress and anxiety.

Participating in physical activity can serve as a constructive outlet for managing stress and anxiety, potentially alleviating symptoms [66]. On the other hand, physical activity can help manage stress and anxiety by releasing endorphins, reducing stress hormones like cortisol, and providing a mindful distraction from worries. It also builds self-esteem and emotional regulation skills, making it a constructive outlet for emotional distress [67]. In our study, we examined whether physical activity changes (moderates) the strength or direction of the relationship between stress and anxiety in adolescents, and found that physical activity reduces the strength of the stress–anxiety link.

In the context of Lazarus and Folkman’s theory of stress and coping, lifestyle factors such as alcohol and nicotine dependence, as well as physical inactivity, may play a significant role as moderators between perceived stress and anxiety [12]. When faced with stress, individuals assess their situations and the resources available to manage them, a process that can be heavily influenced by their lifestyle choices [12]. The present study confirmed that those individuals who are more dependent on substances like alcohol and nicotine may perceive stress as more overwhelming, leading to maladaptive coping mechanisms such as avoidance. This reliance on substances for escape can ultimately exacerbate feelings of anxiety. Similarly, physical inactivity potentially hinders effective coping by limiting access to positive stress-relief strategies, such as exercise, which is known to alleviate stress [24]. As moderators, these lifestyle factors potentially influence the relationship between perceived stress and anxiety: individuals with higher levels of substance dependence experienced increased anxiety in response to stress, while those who maintain a physically active lifestyle reported lower anxiety levels. The findings suggest that perceived stress significantly predicts anxiety levels in both low- and high-physical-activity groups. While the strength of this relationship is generally modest, it is notably stronger among individuals with high physical inactivity compared to those with low physical inactivity. The data also reveal that perceived stress serves as a significant predictor of anxiety across both low- and high-alcohol-dependence groups. However, the strength of this relationship, as indicated by small effect sizes, is notably greater among students with high alcohol dependence compared to those with low alcohol dependence. In addition, our data indicate that, while perceived stress is a significant predictor of anxiety in both low- and high-nicotine-dependence groups, the relationship was stronger (as indicated by medium effect sizes) for students with high nicotine dependence compared to those with low nicotine dependence. Understanding these dynamics is crucial for tailoring interventions that promote healthier coping strategies, thereby mitigating the negative effects of stress on anxiety.

### Strengths and Limitations

The present study integrates multiple psychological and lifestyle factors—stress, anxiety, alcohol and nicotine dependence, physical activity, and social support—allowing for a comprehensive understanding of students’ mental health. By examining multiple psychological and lifestyle variables simultaneously, it offers a holistic view of student mental health. Stratified random sampling ensures the representation and generalizability of the results. Additionally, the research design is ethically sound and relatively easy to implement among student populations. The use of standardized and validated instruments such as the Perceived Stress Scale (PSS-10), Generalized Anxiety Disorder Scale (GAD-7), Alcohol Use Disorders Identification Test (AUDIT), Fagerström Test for Nicotine Dependence (FTND), Multidimensional Scale of Perceived Social Support (MSPSS), and International Physical Activity Questionnaire (IPAQ-short) enhances the reliability and validity of the findings. This study also addresses a relevant public health concern and can inform the development of prevention and intervention programs.

Despite its contributions, the study has several limitations. Being cross-sectional in nature, it can identify associations but cannot establish causal relationships between stress, social support, alcohol and nicotine dependence, physical activity, and anxiety. We also acknowledge that mediation analysis based on cross-sectional data represents a methodological limitation because the causal ordering cannot be empirically established. Future longitudinal research is recommended to explore the temporal relationships among these variables. The reliance on self-report questionnaires may introduce response bias, including social desirability or recall bias, especially when reporting sensitive lifestyle factors such as alcohol use. The study sample may be limited to a specific institution or demographic group, which restricts the generalizability of the findings to broader student populations. Furthermore, unmeasured confounding factors, such as depression, personality traits, or family background, may have influenced the observed relationships. Finally, the study captures participants’ experiences at a single point in time, which may not reflect fluctuations in stress, social support, anxiety, alcohol use, smoking, and physical activity across different academic periods.

## 5. Conclusions

The results of the present study confirm that social support mediates the associations between perceived stress and anxiety among adolescents. This means that social support protects individuals from the adverse effects of stress by providing emotional comfort, practical assistance, and a sense of belonging. When adolescents experience stress, those with higher perceived social support are more likely to appraise stressful situations as manageable and to employ adaptive coping strategies, thereby reducing anxiety. These findings justify programs enhancing social connectedness and support networks (e.g., peer mentoring, counseling, and family engagement), which could reduce anxiety among adolescents. Schools should emphasize emotional education and community building as protective factors against stress-induced anxiety.

Our findings show that lifestyle factors (alcohol and nicotine dependence and physical inactivity) act as moderators between perceived stress and anxiety with significant effects and dampen the associations between perceived stress and anxiety. Adolescents with more nicotine and alcohol dependence, as well as those with a lower level of physical activity, perceive more anxiety when exposed to stress. The deeper analyses of the strength of the moderating effects revealed that the positive relationship between perceived stress and anxiety is stronger (as indicated by medium effect sizes) for students with high nicotine dependence compared to those with low nicotine dependence.

Therefore, the results of our study extend the stress-coping model by identifying negative lifestyle factors as maladaptive moderators that impact anxiety under stress. Our research provides evidence for developing stress-management programs that discourage alcohol use and smoking as a coping mechanism. The results of the present study support Lazarus and Folkman’s theory of stress and coping, showing that lifestyle factors such as alcohol and nicotine dependence, as well as physical inactivity, may play a significant role as maladaptive moderators between perceived stress and anxiety, and also highlight physical activity as a resilience factor in adolescence. Therefore, schools are encouraged to incorporate regular physical activity programs to reduce stress-related anxiety, and educational and health authorities should consider integrating mental health promotion with physical education.

## Figures and Tables

**Table 1:** Descriptives and Cronbach alphas among the investigated variables.

	N = 405		Skewness		Kurtosis		
Scale	*M*	SD	Sk	SkSE	Ku	KuSE	Cronbach α
Anxiety (GAD-7)	10.81	5.11	0.330	0.121	−0.992	0.242	0.94
Perceived stress (PSS-10)	20.91	3.31	0.069	0.121	1.998	0.242	0.92
Perceived social support (MSPSS)	57.50	14.70	−0.196	0.121	−1.132	0.242	0.92
Alcohol dependence (AUDIT)	9.20	7.70	0.202	0.121	−1.538	0.242	0.87
Nicotine dependence (FTND)	2.45	2.99	0.635	0.121	−1.329	0.242	0.76
Physical activity (IPAQ) (log(MET-min/week))	2.94	0.49	−0.238	0.121	−1.766	0.242	0.83

*Notes.* GAD-7 = Generalized Anxiety Disorder Scale; PSS-10 = Perceived Stress Scale; MSPSS = Multidimensional Scale of Perceived Social Support; FTND = Fagerström Test for Nicotine Dependence; AUDIT = Alcohol Use Disorders Identification Test; IPAQ = International Physical Activity Questionnaire. Sk—Skewness; SkSE—Skewness standard error; Ku—Kurtosis. KuSE—Kurtosis standard error.

**Table 2:** Descriptives of gender differences among the investigated variables.

	Men		Women			
	N = 149		N = 256			
Scale	*M*	SD	*M*	SD	*t*	d
Anxiety (GAD-7)	12.62	5.49	9.75	4.56	5.64 ***	0.58
Perceived stress (PSS-10)	21.25	3.15	20.63	3.38	1.81	0.19
Perceived social support (MSPSS)	52.26	15.17	60.55	13.55	−5.68 ***	−0.59
Alcohol dependence (AUDIT)	12.42	8.13	7.32	6.79	6.76 ***	0.69
Nicotine dependence (FTND)	3.60	3.26	1.77	2.60	6.22 ***	0.64
Physical activity (IPAQ) (log(MET-min/week))	2.09	1.40	1.22	1.49	5.87 ***	0.61

*Notes. p* calculated in Student’s *t*-test. *** *p* < 0.001 (two-tailed). d—Cohen’s d. GAD-7 = Generalized Anxiety Disorder Scale; PSS-10 = Perceived Stress Scale; MSPSS = Multidimensional Scale of Perceived Social Support; FTND = Fagerström Test for Nicotine Dependence; AUDIT = Alcohol Use Disorders Identification Test; IPAQ = International Physical Activity Questionnaire.

**Table 3:** Pearson correlations between gender, psychosocial factors, and lifestyle components.

	GAD-7	PSS-10	MSPSS	AUDIT	FTND	IPAQ	Gender
Anxiety (GAD-7)	1						
Perceived stress (PSS-10)	0.486 **	1					
Social support (MSPSS)	−0.777 **	−0.362 **	1				
Alcohol dependence (AUDIT)	0.773 **	0.359 **	−0.791 **	1			
Nicotine dependence (FTND)	0.693 **	0.334 **	−0.733 **	0.792 **	1		
Physical activity (IPAQ)	−0.284 **	−0.035	0.305 **	−0.318 **	−0.276 **	1	
Gender	−0.271 **	−0.090 *	0.272 **	−0.319 **	−0.296 **	0.098 *	1

*Notes.* Total sample N = 405; ** *p* < 0.01 (two-tailed); * *p* < 0.05 (two-tailed). GAD-7 = Generalized Anxiety Disorder Scale; PSS-10 = Perceived Stress Scale; MSPSS = Multidimensional Scale of Perceived Social Support; FTND = Fagerström Test for Nicotine Dependence; AUDIT = Alcohol Use Disorders Identification Test; IPAQ = International Physical Activity Questionnaire.

**Table 4:** Results of multiple linear regression analysis predicting anxiety (GAD-7) (n = 405).

	Model Variables	*B*	SE(*B*)	β	*t*	*p*	R^2^	ΔR^2^	Lower 95% CI	Upper 95% CI
Block 1	Gender	−0.242	0.447	−0.229	−5.414	<0.001			−3.300	−1.542
	Perceived stress (PSS-10)	0.719	0.065	0.466	11.017	<0.001	0.288		0.591	0.847
Block 2	Gender	−0.698	0.322	−0.066	−2.167	0.031			−1.331	−0.065
	Perceived stress (PSS-10)	0.365	0.049	0.236	7.515	<0.001			0.269	0.460
	Social support (MSPSS)	−0.234	0.011	−0.673	−20.703	<0.001	0.656	0.368 ***	−0.256	−0.212
Block 3	Gender	−0.278	0.306	−0.026	−0.911	0.363			−0.879	0.322
	Perceived stress (PSS-10)	0.321	0.046	0.208	7.035	<0.001			0.231	0.411
	Social support (MSPSS)	−0.137	0.016	−0.394	−8.351	<0.001	0.701	0.47 ***	−0.169	−0.105
	Alcohol dependence (AUDIT)	0.246	0.032	0.371	7.748	<0.001	0.701	0.47 ***	0.184	0.308
Block 4	Gender	−0.238	0.305	−0.022	−0.779	0.436			−0.828	0.371
	Perceived stress (PSS-10)	0.322	0.046	0.209	7.042	<0.001			0.233	0.413
	Social support (MSPSS)	−0.127	0.017	−0.364	−7.503	<0.001			−0.160	−0.093
	Alcohol dependence (AUDIT)	0.205	0.036	0.308	5.655	<0.001			0.134	0.276
	Nicotine dependence (FTND)	0.157	0.079	0.092	1.973	0.049 *			0.001	0.313
	Physical activity (IPAQ)	−0.659	0.295	−0.069	−2.239	0.026 *	0.705	0.004 *	−1.239	−0.080

*Notes.* β represents the standardized regression coefficients for each block in the regression equation. * *p* < 0.05; *** *p* < 0.001. CI = Confidence interval based on 1000 bootstrap samples for *B*.

**Table 5:** Indirect and direct effects and 95% confidence intervals (CIs) for the mediational analysis in the relationship between perceived stress and anxiety (n = 405).

Type	Model Variables	*B*	SE(*B*)	Lower 95% CI	Upper 95% CI	β	*z*	*p*
Indirect	PSS-10 ⇒ MSPSS ⇒ GAD-7	0.387	0.0525	0.284	0.490	0.251	7.37	<0.001
Component	PSS-10 ⇒ MSPSS	−1.609	0.2058	−2.013	−1.206	−0.362	−7.82	<0.001
	MSPSS ⇒ GAD-7	−0.240	0.0109	−0.262	−0.219	−0.692	−21.99	<0.001
Direct	PSS-10 ⇒ GAD-7	0.364	0.0486	0.268	0.459	0.236	7.49	<0.001
Total	PSS-10 ⇒ GAD-7	0.751	0.0671	0.619	0.882	0.486	11.18	<0.001

*Notes.* GAD-7 = Generalized Anxiety Disorder Scale; PSS-10 = Perceived Stress Scale; MSPSS = Multidimensional Scale of Perceived Social Support. CI = Confidence interval based on 1000 bootstrap samples for *B*.

**Table 6:** Moderation regression analyses, perceived stress, and lifestyle predicting anxiety (GAD-7) (n = 405).

	Model Variables	β	*t*	*p*	R^2^	ΔR^2^	Lower 95% CI	Upper 95% CI
Block 1	Perceived stress (PSS10)	0.463	11.704	<0.001			0.594	0.834
	Physical activity (IPAQ)	−0.373	−9.432	<0.001	0.375	0.138 ***	−4.302	−2.818
Block: 2	Perceived stress (PSS10)	0.464	11.873	<0.001			0.598	0.835
	Physical activity (IPAQ)	−0.372	−9.531	<0.001			−4.291	−2.824
	Interaction Z_Physical activity × Z_Perceived stress	−0.124	−3.171	0.002	0.390	0.015 **	−0.208	−0.888
Block 1	Perceived stress (PSS10)	0.288	9.238	<0.001			0.350	0.540
	Alcohol dependence (AUDIT)	0.668	21.420	<0.001	0.643	0.076 ***	3.831	4.605
Block 2	Perceived stress (PSS10)	0.287	9.273	<0.001			0.350	0.540
	Alcohol dependence (AUDIT)	0.682	21.637	<0.001			3.912	4.694
	Interaction Z_Alcohol dependence (AUDIT) × Z_Perceived stress	0.073	2.422	0.016	0.648	0.005 *	0.071	0.686
Block 1	Perceived stress (PSS10)	0.303	8.400	<0.001			0.358	0.577
	Nicotine dependence (FTND)	0.573	15.896	<0.001	0.531	0.082 ***	2.634	3.378
Block 2	Perceived stress (PSS10)	0.279	7.694	<0.001			0.321	0.541
	Nicotine dependence (FTND)	0.607	16,420	<0.001			2.802	3.565
	Interaction Z_Nicotine dependence (FTND) × Z_Perceived stress	0.119	3.390	<0.001	0.544	0.013 **	0.088	0.330

*Notes.* β represents the standardized regression coefficients for each block in the regression equation. * *p* < 0.05; ** *p* < 0.01; *** *p* < 0.001. GAD-7 = Generalized Anxiety Disorder Scale; PSS10 = Perceived Stress Scale; FTND = Fagerström Test for Nicotine Dependence; AUDIT = Alcohol Use Disorders Identification Test; IPAQ = International Physical Activity Questionnaire. “Z” indicates that the variable has been standardized (converted to z-scores). CI = Confidence interval based on 1000 bootstrap samples for *B*.

## Data Availability

The datasets collected and analyzed during the current study are available from the corresponding author on request.
